# An Energy-Efficient and Secure Data Inference Framework for Internet of Health Things: A Pilot Study

**DOI:** 10.3390/s21010312

**Published:** 2021-01-05

**Authors:** James Jin Kang, Mahdi Dibaei, Gang Luo, Wencheng Yang, Paul Haskell-Dowland, Xi Zheng

**Affiliations:** 1 School of Science, Edith Cowan University, Joondalup 6027, Australia; w.yang@ecu.edu.au (W.Y.); p.haskelldowland@ecu.edu.au (P.H.-D.); 2 Department of Computer Engineering, Islamic Azad University Tabriz Branch, Tabriz 5166616471, Iran; dibayimahdi@yahoo.com; 3 Department of Computing, Macquarie University, Sydney 2109, Australia; gangluo96@gmail.com

**Keywords:** privacy-preserving, body sensors, wireless body area network (WBAN), Internet of Health Things (IoHT), mHealth, IoT, cloud, healthcare big data, inference system

## Abstract

Privacy protection in electronic healthcare applications is an important consideration, due to the sensitive nature of personal health data. Internet of Health Things (IoHT) networks that are used within a healthcare setting have unique challenges and security requirements (integrity, authentication, privacy, and availability) that must also be balanced with the need to maintain efficiency in order to conserve battery power, which can be a significant limitation in IoHT devices and networks. Data are usually transferred without undergoing filtering or optimization, and this traffic can overload sensors and cause rapid battery consumption when interacting with IoHT networks. This poses certain restrictions on the practical implementation of these devices. In order to address these issues, this paper proposes a privacy-preserving two-tier data inference framework solution that conserves battery consumption by inferring the sensed data and reducing data size for transmission, while also protecting sensitive data from leakage to adversaries. The results from experimental evaluations on efficiency and privacy show the validity of the proposed scheme, as well as significant data savings without compromising data transmission accuracy, which contributes to energy efficiency of IoHT sensor devices.

## 1. Introduction

The release of contact tracing applications in response to the COVID-19 pandemic has highlighted some of the vulnerabilities and potential privacy issues that can be associated with these applications. Private information can be vulnerable to being compromised if communication protocols with weak security are used in such applications, such as Bluetooth, which was used by some health agencies [1]. Smart home environments that are integrated with health applications are becoming increasingly prevalent as more homes are being connected to the Internet of Things (IoT) and Internet of Health Things (IoHT) networks, along with wearable devices. As the demand for these services increase, additional data transactions and network activity will increase the workload of wireless body area networks (WBAN), which consist largely of sensors and smartphones. These devices, such as physiological sensors and monitoring devices, will be affected by an increased demand in performance and battery power. The current capabilities of sensor technologies limit their interaction with IoT networks and are yet to have the intelligence [2] to securely provide data to health networks. Rather, these devices have a more passive function and only provide data at a regular interval or on an on-demand basis due to their hardware size and battery limitations. The use of some devices, such as smartphones, to interact with sensors and wearables makes it possible to overcome some of these limitations by taking advantage of the more powerful resources that are provided by smartphones. Despite this ability to access additional resources from more powerful devices, energy efficiency remains a fundamental requirement in considering IoHT network design, given that a large number of IoHT devices are powered by batteries that have a limited lifespan. In addition, privacy is another requirement for handling health information. Privacy is often defined as having the ability to protect sensitive data, such as personal information. It is expected that, as connectivity to IoHT increases, the volume of traffic and transactions of data requests to sensors in IoHT networks will increase [3]. With the increasing volume of health data being collected and utilized across multiple devices in IoHT networks, user privacy is at greater risk if health data are not securely protected. Because the field of sensor and IoHT device interactions is a novel and emerging area, there remains a scarcity of research that addresses the privacy requirements of sensitive health data within such a context. Securely transferring health data across these networks whilst maintaining user privacy poses a difficult challenge to address.

**Figure 1:**
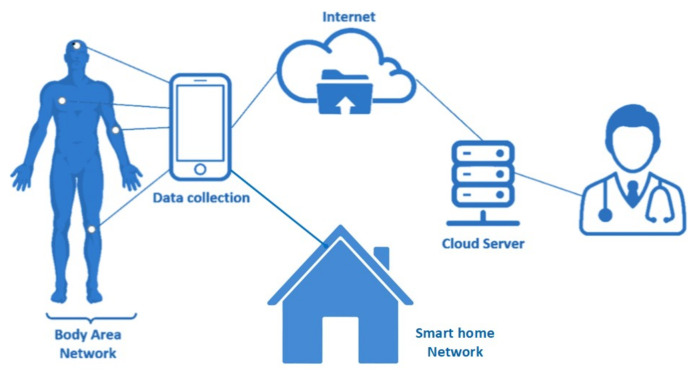
Smart home network merged with a healthcare Internet of Health Things (IoHT) network model.

Novel approaches are needed in order to achieve both energy efficiency and privacy preservation when designing IoHT networks. In this paper, we propose an energy-efficient and secure data inference framework for IoHT applications, e.g., a smart house health care system (as demonstrated in Figure 1), which enables the collected sensitive information from the smart house health care system to be transferred in the encrypted domain while simultaneously reducing the energy consumption. The proposed framework involves two tiers, which consist of the data reduction tier and data protection tier. This two-tier approach is specifically designed for IoHT applications, where privacy in the underlying sensor data is protected by a privacy-preserving workflow. In these applications, the sensor data are first reduced, and the encrypted sensor data are then transmitted to edge servers. At the edge server, differential privacy is used to further protect privacy. In more detail, the first tier infers the data processing of sensors to reduce transactions from sensors to smartphones and IoHT networks. Processed encrypted data from wearable devices will be passed to the second tier. The second tier protects data by Laplace noise enabled differential privacy in order to protect the privacy of each user. Three major contributions to the field can be delineated from this proposal:

- Leveraging model driven prediction, encryption, and data points (DP) with edge computing to propose a two-tier privacy-preserving IoHT framework that does not currently exist.
- Evaluation of the proposed system in terms of efficiency and privacy preservation with up to 98.83% and 95.95% of data savings rate (SR) and accuracy rate (AR), respectively, while maintaining sufficient accuracy that is arbitrarily required by users.
- Presenting potential application scenarios that would benefit from this solution.

This work is a significant extension of a previously published study [4], which initially outlined the proposed concept. When compared with that study, this paper adds three key differences in terms of the solution, experiment, and motivation applications. Specifically, (1) solution: greater attention has been focused on the first tier of the solution by developing new formulations for AR and SR. (2) Experiment: regarding the new formulation, new experiments have been undertaken with different values of evaluation metrics to calculate the AR and SR. (3) Application: several potential applications of the proposed framework are listed. In view of the energy efficiency and privacy preservation concepts in this framework, a small number of beneficial applications have been examined, including patient monitoring during a pandemic, the battery conservation of personal health devices (PHD), and the use of biometrics for remote identification.

In the remainder of this paper, Section 2 reviews the existing related literature. Section 3 presents, in detail, the two-tier privacy-preserving data inference framework proposal. Section 4 discusses the results of efficiency and security analysis of the proposed system. Section 5 describes possible applications for which the proposed framework could be implemented. Section 6 finishes the paper with some concluding remarks.

## 2. Related Works

An IoHT network is defined as an IoT network that includes a PHD, which itself is defined in further detail by the IEEE P11073 PHD Work Group. IoHT could include any health devices that are attached on or within a user’s body, and these are predominantly battery driven with the ability to sense certain health or physiological data. It should also have memory storage capacities and be capable of wireless communication. In order to assess the proposed solution with PHD devices and networks, related areas are reviewed, including inference systems and privacy-preservation techniques.

### 2.1. WBAN and IoT Networks

WBANs and IoHT networks provide interfaces to a cloud monitoring centre, where health data can be transmitted for processing and retrieval by health practitioners and other users. Some interfaces between IoHT and electronic Health (eHealth) networks have sufficient resources available in terms of power and system capacity, as they are fixed devices with a permanent power source, such as Computed Tomography (CT) machines. These do not rely on portable powered devices, such as pacemakers implanted inside a patient and powered by an internal battery. This differentiates the mHealth system from the overall eHealth system and it presents its own unique challenges.

Some related works have addressed the improvement of sensor networks to process data, with solutions, such as using middleware in a new global sensor network infrastructure, improving routing protocols [5,6,7], or the acquisition of reading and modelling the accuracy of sensors using algorithms [8]. However, these have not attempted to minimize data sampling and transmission from sensors. Bragg et al. [9] have stated that, as hospitals grow in densely populated cities, the transmission of large amounts of data in hospital wireless networks becomes a crucial problem. The proposed method leverages a reinforcement learning protocol to consider scheduling priority for data transmission in WBANs based on data criticality and deadlines. The network architecture consists of biosensors in wearable devices (e.g., wrist-worn piezoelectric-based sensors for blood pressure monitoring) and implanted devices (e.g., pacemakers, neurostimulators) that communicate wirelessly with a patient data controller (PDC). The PDC is also connected to headquarters in the hospital. When a packet arrives at a PDC, it is placed into a queue and scheduled to be transmitted to headquarters by a reinforcement learning protocol. The reinforcement learning protocol consists of a set of states, a set of actions, cost function, and value function. The proposed solution overlooks the fact that not every DP has to be transferred if it is redundant or does not carry any meaning of new significance when compared to the subsequent or previous DPs before it. In fact, sending all the available data increases bandwidth and energy usage. It is worth mentioning that IEEE PHD P11073-20601 [10] specifies security protocols that are to be used in PHDs during message exchange while using the concept of ‘agents’ and ‘managers’. Layers with transport independence, such as Bluetooth for health device profiles, Universal Serial Bus (USB) for the personal healthcare device class, and ZigBee for health care profiles, are recommended by IEEE 11073.

### 2.2. Health Inference and Prediction Analysis

Overhead requirements can be reduced by inferring health data on sensor devices, which circumvents the need to have a managing device, such as a smartphone or other smart device, to act as a gateway that connects to a public network. Engel et al. [11] have proposed an inference model for the continuous monitoring of goods in supply chain management in order to provide relevant information for users. The authors have argued that RFID and multi-sensor wireless sensor networks (WSNs) that are used in logistics applications produce a huge volume of data. The context-aware inference model makes use of available computational intelligence models, including supervised learning, unsupervised learning, rule-based models, fuzzy logic, ontology-based models, or probabilistic logic, to obtain relevant information from processed data. Although the proposed context-aware inference system enhances performance and efficiency in logistics operations, inferencing within situations where data traffic requests originate from an external party (e.g., IoHT) have not been considered. Zhu et al. [12] utilized the dynamic Bayesian model averaging in order to predict the occurrence of future events and a number of future states based on previously monitored events in IoT. However, the size and type of health-related data in mHealth data collections are different from that of IoT applications. Therefore, this model is not appropriate for mHealth data. Ljaz et al. [13] solved the latency and overloading problems in smart healthcare applications by developing a tri-fog health architecture, which includes three layers, consisting of a wearable layer, intelligent fog layer, and cloud layer. Pazienza et al. [14] explored the machine learning technique to find the most suitable machine learning algorithm for predicting the clinical risk classes of patients.

### 2.3. Privacy Preservation

Privacy-preservation is critical in many networks, such as the cloud, WSNs, and, especially, in the eHealth environment. There are security and privacy aspects to be considered when transmitting health data to any network. The following presents an overview of two types of existing privacy-preserving technologies that are relevant to this study.

#### 2.3.1. Cryptography-Based Schemes

Encryption can be defined as an ordered quintet (*P*, *C*, *K*, *E*, *D*), where *P* is the *plaintexts*, *C* is the *crypto texts*, *K* is the *keys*, *E* is the *encryption function,* and *D* is the *decryption function*. Pasupuleti et al. [15] proposed a secure privacy-preserving scheme based on probabilistic public key encryption algorithm for securing outsourced data of resource-limited mobile devices. In order to reduce the computation and communication overhead, the proposed system uses a ranked keyword search, which first returns the most relevant files instead of all files. Wang et al. proposed a hierarchy attribute-based encryption scheme to secure the shared data using cipher text-policy attribute-based encryption. An integrated access structure, together with some attributes, are involved in the encryption. The proposed scheme has been proven to be efficient with the increment of the number of files [16]. Wasters [17] proposed an attribute-based encryption method. In his solution, it allows for the data sender to determine the access control policies. A user can only decrypt the cipher text when the access tree that is associated with that cipher text is satisfied by the attribute set, which is associated with the private key.

#### 2.3.2. Differential Privacy-Based Schemes

A major problem with sharing information about a dataset is privacy preservation. Differential privacy is a technique for modifying data in a way that prevents inferring much about private information. Yin et al. [18] proposed a location privacy-preserving scheme that is based on the differential privacy strategy for IoT networks. A location information tree model is constructed in order to express the position dataset. The authors claimed that the proposed scheme could achieve higher processing efficiency when compared to traditional location privacy protection algorithms. Another privacy-preserving framework for exchanging gradients in federated learning with chained secure multi-part computing technique is proposed in Li et al. [19]. The authors argued that the proposed approach is equivalent to differential privacy when *E =* 0, which prevents sensitive information leakage (e.g., gradients). Xu et al. [20] proposed a framework for IoT data analysis, called local differential privacy obfuscation, which can ensure that users’ sensitive data will not be exposed when they are aggregated and distilled at the IoT devices. Liu et al. [21] proposed a framework for directing traffic flow from one smart home to another home gateway prior to sending to the internet, which achieves strong differential privacy and prevents attackers from linking the traffic flow back to a specific smart home network.

## 3. The Proposed Solution

We propose a privacy-preserving two-tier data inference framework in order to reduce the power consumption of IoHT devices and protect sensitive health data generated by IoHT networks, which has an assumption of low data rates for transmission. The first tier in this framework involves a data inference algorithm that can reduce the number of redundant or low-value transactions to save power consumption; the second tier protects the sensitive data using encryption and differential privacy techniques. Because most battery power consumption occurs with data transmission over radio from sensors to a smart device, which collects sensed data and transfers them to a server in the cloud, the reduction of the frequency of data transmission at the source nodes are crucial in saving and conserving battery power, which is achieved by the inferencing algorithm at the source nodes. Improving accuracy and efficiency at the source node is achieved by improving an inference algorithm, which provides energy efficiency and is critical in contributing to the security of IoHT networks. The proposed two-tier data inference framework provides enhanced accuracy and efficiency when compared to the existing single layer inference algorithm [22].

### 3.1. The First Tier Data Reduction Using a Data Inference Algorithm

It is unnecessary to consume bandwidth and power resources by sending all available data if there could be a more effective method for reducing the volume of the original data sent. Therefore, in the first tier, it is proposed to use a data inference algorithm, which only decides to transmit data if they are significantly different from previously captured DPs, thus reducing the number of redundant or low-value data transfers [23]. Using this solution, there is a risk of reducing accuracy from the original data and that it may not properly represent data in certain situations, such as in the case of short interval sampling. To reduce these instances, it is proposed to analyze the differences between the original and inferred data and apply regular beacons (DPs, which are transmitted regardless) into the inferred results, such that they are transmitted regularly to roughly reflect the original data and can improve the accuracy when augmented with the inferred DPs. Three aspects are considered to assess the results [22], including: (1) Efficiency Ratio (ER) of saved (reduced) data volume and actual transmitted data, (2) Savings Ratio (SR) of reduced data and sensed data (%), and (3) Accuracy Ratio (AR) of total value of transmitted data and original data (%) [22].

(1) $$\mathrm{Savings}\;\mathrm{Rate}\;\left(\mathrm{SR}\right)\;=\frac{\mathrm{No}\;\mathrm{of}\;\mathrm{Sensed}\;\mathrm{data}-\mathrm{No}\;\mathrm{of}\;\mathrm{Transferred}\;\mathrm{data}}{\mathrm{Number}\;\mathrm{of}\;\mathrm{Sensed}\;\mathrm{data}}\times 100$$

(2) $$\mathrm{Efficiency}\;\mathrm{Rate}\;\left(\mathrm{ER}\right)=\;\frac{1}{1-\frac{\mathrm{SR}}{100}}$$

(3) $$\mathrm{Accuracy}\;\mathrm{Rate}\;\left(\mathrm{AR}\right)\;=\frac{\mathrm{Sum}\;\mathrm{of}\;\mathrm{original}\;\mathrm{DPs}-\;\mathrm{Sum}\;\mathrm{of}\;\mathrm{differences}\;}{\mathrm{Sum}\;\mathrm{of}\;\mathrm{original}\;\mathrm{DPs}}\times 100$$

Variance rate (VR) is used for inferring the selection and subsequent transmission of data. It compares the DP with those directly before and afterwards to screen out DPs that are too similar and do not have to be transmitted, i.e., DPs that do not provide new significant information from previous DPs. Different levels of granularity can be applied for VRs, e.g., 1% VR is finer than 10% VR. It can be applied while using the Algorithm 1 below.
**Algorithm 1:** Variance rate algorithm initialization;1:**Initialization**2:**if** |*Vc* − *Vc*_1_|*OR*|*Vc*_0_ − *Vc*| *> Vc* ∗ *Vr* **then**3:*Vx ← Vc*;4:**else**5:*Vx ← null*;
where *Vc* is current value, *Vc*_0_ is previous value, *Vc*_1_ is next value, *Vx* is sampling value, and *Vr* is variance rate.

**Figure 2:**
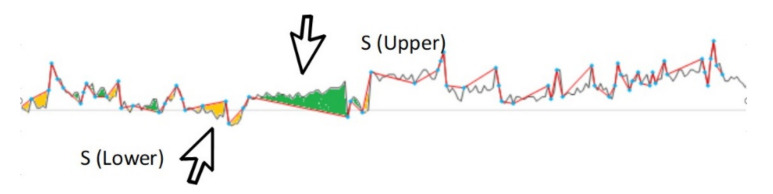
Depiction of gaps between original data points (DPs) and inferred DPs to show the accuracy of the calculation [20].

When a VR is applied to data, a difference between the graphs of inferred data versus the graph of original data will inevitably arise, as depicted in Figure 2. In this figure, S (Upper) represents the area of this difference or the distorted portion by the inferred values that are less than the original, whilst S (Lower) represents the areas of inferred values that are higher than the original. A larger total area of the gap refers to greater data distortion and, therefore, reducing this gap would imply better accuracy. The formula below depicts the area of upper and lower sides of the inferred graph against the original.

(4) $$S_{u}=\sum_{k=0}^{n}(\begin{array}{c} n\\ k \end{array})S_{n}{}^{},\;where\;S_{n}\;=\;G\;\left(S1,\;S2,\;\ldots ,\;Sn\right)$$

Similarly

(5) $$S_{l}=\sum_{k=0}^{n}\left(\begin{array}{c} n\\ k \end{array}\right)S_{n}{}^{},\;where\;S_{n}\;=\;Y\;\left(S1,\;S2,\;\ldots ,\;Sn\right)$$

The total area of the gaps would be presented, as below. A larger value means a ‘coarser’ and higher VR inference has been used relative to a smaller total area, which means that a ‘finer’ and lower VR value has been applied.

The larger the difference ($S_{d}$ = |$S_{u}$*−*$S_{l}$|), the further the result is from the average and, hence, from the original trend. However, it is important to note that a smaller difference does not necessarily mean that it represents the original data graph properly—it could, however, be an indicator of how accurate the inference is to the original, along with the gaps instead. For example, a small S value as well as a small $S_{d}$ suggests that it is likely to be closer to the original. These figures in conjunction (i.e., *S* and $S_{d}$) can be used to determine how accurate each inference is, whilst the savings or the reduction of DP indicate the efficiency.

(6) $$S=\;S_{u}+\;\;S_{l}$$

The following formulations can represent the accuracy rate and savings rate:

(7) $$AR=\;\frac{\sum original\;DPs-\;\sum S_{d}}{\sum original\;DPs}\;\times \;100\%$$

(8) $$SR=\;\frac{DPs-N\left(DPs\right)}{DPs}\;\times \;100\%$$

where *DPs* are the number of sensed data points and *N (DPs)* is the number of *DPs* after inference.

When *S* = 0, it would suggest that the inference represents the original data perfectly with no distortion, whilst a $S_{d}$ = 0 suggests that the inference represents the mean value of the graph, despite not perfectly representing the original. Figure 2 depicts the upper and lower gaps after inferencing has been applied. The reduction of *DPs* is a consequence of sampling in statistical inference systems, which leads to data size reduction. However, when increasing the VR results in increased savings, there should be a threshold to ensure accuracy of the result. Privacy preservation is increasingly being recognized as a serious concern for IoHT networks where healthcare data are shared, processed, and transferred. The degree of privacy preservation in inference systems would be described in what extent that inferred data are different from the original data. The sampled data will then be encrypted while using symmetric key encryption (SKE), or attribute-based encryption (ABE). As an explanation, ABE is a public key encryption (PKE) technique [24]. The encrypted data are then passed to the second tier.

### 3.2. The Second Tier Data Protection with Differential Privacy

The second tier concerns the protection of sensitive health data that were created by the IoHT network in the first tier. In order to protect the privacy of sensitive data in the dataset, removing identifying and personal information, such as the user’s name, ID, and phone number, is insufficient, because the remaining data reveal identities in the dataset. Differential privacy is a technique that ensures protection against attackers to infer private information [25]. In the differential privacy algorithms, a randomized function adds a random noise to the true answer in order to produce a response to a query [26].

#### Definition of Differential Privacy

Let *D* and *D*’ be two neighbouring datasets and *M* a randomized function. *M* provides E-differential privacy for all sets of O ⊆ Range (M), if it satisfies the following:

(9) $$\frac{\mathrm{Pr}\left[\;M\left(D\right)\;\in O\;\right]}{\mathrm{Pr}\left[\;M\left(D^{\prime }\right)\;\in O\right]}\leq \exp\left(\epsilon \right)$$

It is said that algorithm *M* provides E-differential privacy protection. It can be seen from the definition of differential privacy that the E is used in order to control the probability ratio of the algorithm *M* to obtain the same output on two adjacent data sets. It reflects the level of privacy protection that *M* can provide. In practical applications, E usually takes a small value, such as 0.01, 0.1, or 1n 2, 1n 3. The value of E should be combined with specific requirements to achieve a balance of safety and the availability of output results. Differential privacy protection can be achieved by adding an appropriate amount of interference noise to the return value of the query function. Adding too much noise will affect the usability of the result, while too little cannot provide sufficient security. Sensitivity is a key parameter that determines the amount of noise that is added. It refers to the largest change to the query result that is caused by adding or deleting any record in the data set.

For $f:\;D\to R^{d}$, the L1-sensitivity of f is

(10) $$\Delta f=\underset{D_{1},\;D_{2}}{\max}||f\left(D_{1}\right)-f\left(D_{2}\right)||{\;}_{1}$$

for all $D_{1}$, $D_{2}$ differing in one element at most.

The sensitivity of a function is determined by the function itself, and different functions will have different sensitivities. For functions with lower sensitivity, sufficient privacy protection can be achieved with the addition of only a small amount of noise. However, for some sensitive functions (such as the median function), it is required to add a lot of noise in order to achieve the same level of protection.

Laplace Mechanism and Exponential Mechanism are the most common implementation mechanisms. Probability Density Function (PDF) for a random variable with Laplace distribution is defined, as follows:

(11) $$Laplace\left(x|\;µ,b\right)=\frac{1}{2b}exp(-\frac{\left|x-µ\right|}{b})$$

Let $b=\frac{\Delta f}{\epsilon }$ where *f* is the query function. Then, we have

(12) $$Laplace\left(x|\;µ,\;\epsilon ,\;\Delta f\right)=\frac{\epsilon }{2\Delta f}exp(-\epsilon \frac{\left|x-µ\right|}{\Delta f})$$

## 4. Results and Analysis

### 4.1. Efficiency and Accuracy Evaluation

**Figure 3:**
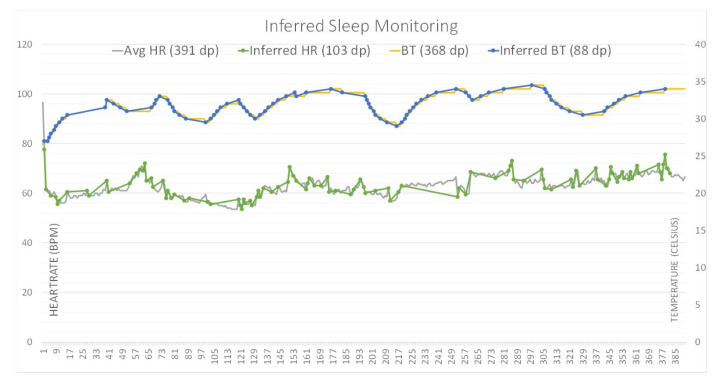
Inferred heart rate (HR) and body temperature (BT) of sleep monitoring data (based on sampled by minutes)–inferred BT data represents the original well whilst inferred HR data shows relatively more gaps (this could be improved by using beacon data sampling).

The approach that is used for evaluation has heart rate (HR) samples, whilst other variables could also be used, such as skin temperature, blood pressure, respiration rate, and indicators of specific diseases, such as diabetes, plethora signals, etc. Because the aim of this experiment is to investigate and evaluate health data efficiency and accuracy, the algorithm created is focused on HR data, which can respond relatively quickly to the user’s activity and time. Body temperature hardly varies or fluctuates in response to changes as the human body automatically maintains its value within a tight range, as shown in Figure 3. Therefore, HR data were primarily measured and used in the experiment.

Fine and coarse inference algorithms were applied to show the differences and efficiency of each of these cases. Dataset [22] was used for HR and BT with Matlab R2019b for the inference algorithm.

The evaluation results are displayed in Figure 3 and they depict body temperature (BT) and heart rate (HR) sensed on a per-minute basis. In applying a 1% and 2.5% inference rate to BT and HR data, respectively, the volume of data to be transferred was reduced by 76% for BT and 73% for HR.

BT inference shows better results, representing almost identical data as opposed to HR. In other words, whilst the data savings rates are similar for both BT and HR, the accuracy of inference in both types of data were very different and they could be reflective of the inherent differences in what these data are measuring. Distortion of the original data can occur when an inference system disregards data to transmit if it does not vary sufficiently from previous or adjacent DPs, i.e., does not meet a stated VR threshold and is, therefore, determined to be of little significance and considered as unnecessary for transmission. This distortion is especially so for data that are measured at shorter intervals, as the data could be trending over a longer term, but simply due to the shorter frequency of data measurements, do not have time to vary significantly between each subsequent data measurement. This limitation was discussed in detail earlier in Section 3, along with a potential solution, which is to add DPs that function as beacons. These beacons transmit data at set intervals, regardless of whether they meet the VR threshold criteria and, therefore, helps to maintain the accuracy of the overall inference data without heavily compromising on data savings. In these experiments, beacon DPs were set to minute intervals. A finer inference VR threshold can provide greater accuracy; however, it results in lesser transmission savings and decreases the overall efficiency rate from the perspective of data transmission. Certain situations may simply require a general idea of the trend, rather than valuing exact or accurate figures—in these cases, a coarser inference VR method could be used instead, which places greater priority on data saving. The exact interval of beacon DPs would depend on the context and solution or application requirements for which this inference is being implemented.

The evaluation of efficiency and accuracy of the proposed inference system has been extended while using series 1 and series 2 of the heart rate time series dataset [27]. Each series contains 1800 evenly spaced measurements of the instantaneous heart rate from a single subject. The extension of the experiments has been done in nine cases, as follows:

**Case 1**: the processing method of case 1 on the original data set is to remove the same DPs in the data set. For example, when the three adjacent DPs and their corresponding value are DP1 = 84.7, DP2 = 84.7, DP3 = 84.7, only DP1 will be retained, while DP2 and DP3 will be discarded.

The original data set has 1800 DPs, as shown in Table 1. After removing consecutive similar points, the data set is left with 1716 DPs i.e., 1800 − 1716 = 84 points are reduced. When using the method of removing the same data for inference, according to the Equations (4) and (5), SR = (84/1800) × 100% = 4.67%; at the same time, AR = 99.74%.

**Case 2**: it takes part of the data from the original data set as an output by time sampling. The sampling rate of the original data set used in this experiment was 0.5 s. When executing case 2, we set to extract one data point from the original data every 30 s as the output. That is, the (30 × N)/0.5, (N = 1, 2, 3, 4,...) points of the original data were used.

The original data set has 1800 DPs, as can be seen in Table 1. After sampling at 30 s intervals, there were 31 DPs left in the data set, which means a reduction of 1800 − 31 = 1769 points. According to Equations (4) and (5), SR = (1769/1800) × 100% = 98.27%; at the same time, AR = 96.26%.

**Case 3**: in the same vein, case 3 takes part of the data from the original data set as an output by time sampling. The sampling rate of the original data set in this experiment was 0.5 s. However, in this case, we set to extract one data point from the original data every 60 s as the output. That is, the (60 × N)/0.5, (N = 1, 2, 3, 4,...) points of the original data were used as the output.

The original data set has 1800 DPs, as shown in Table 1. After sampling at 60 s intervals, there were 16 DPs left in the data set, which means a reduction of 1800 − 16 = 1784 points. Using Equations (4) and (5), SR is equal to 99.11% and AR is equal to 95.73%.

**Case 4**: in case 4, the sampling rate of the original data set was again 0.5 s. However, one data point was extracted from the original data every 120 s. Therefore, the (120 × N)/0.5, (N = 1, 2, 3, 4,...) points of the original data were used.

The original data set has 1800 DPs, as shown in Table 1. After sampling at 120 s intervals, there were nine DPs left in the data set, which means a reduction of 1800 − 9 = 1791 points

According to the Equations (4) and (5), SR is equal to 99.50% and AR is equal to 94.18%.

**Case 5**: similar to previous cases, the sampling rate was 0.5 s in this case. One data point is extracted from the original data every 180 s while using (180 × N)/0.5, (N = 1, 2, 3, 4,...) points of the original data.

After sampling at 180 s intervals, there were six DPs left in the data set (as shown in Table 1), which means a reduction of 1800 − 6 = 1794 points. SR and AR can be calculated by Equations (4) and (5). According these formulations, SR = (1794/1800) × 100% = 99.66% and AR = 94.21%.

**Case 6**: in case 6, the first step was to obtain intermediate data 1 using VR inference with VR = 2%. The second step was to obtain the intermediate data set 2 after sampling with a sampling interval of 60 s. The third step was to merge the intermediate data set 1 and the intermediate data set 2 in order to obtain the final output.

The original data set has 1800 DPs, according to the Table 1. After the inference of VR = 2% and 60 s interval sampling, there were 182 DPs left in the data set. In other words, 1800 − 182 = 1618 points were reduced. Meanwhile, SR = (1618/1800) × 100% = 89.88% and AR = 97.57%.

**Case 7**: the first step was to obtain intermediate data 1 by using VR inference with VR = 3%. The second step was to obtain the intermediate data set 2 after sampling with a sampling interval of 60 s. The third step was to merge the intermediate data set 1 and the intermediate data set 2 to obtain the final output.

The original data set has 1800 DPs, as shown in Table 1. After the inferencing of VR = 3% and 60 s interval sampling, there were 64 DPs left in the data set. In other words, 1800 − 64 = 1736 points were reduced. Using Equations (4) and (5), SR is equal to (1736/1800) × 100% = 96.44% and AR is equal to 96.04%.

**Case 8**: this is similar to case 7. When compared with case 7, the difference is that, in case 8, VR is equal to 10%. After the inferencing of VR = 10% and 60 s interval sampling, there were 22 DPs left in the data set. In other words, 1800 − 22 = 1778 points were reduced. According to Equations (4) and (5), SR = (1778/1800) × 100% = 98.78% and AR = 96.14%.

**Case 9**: in case 9, the first step was to obtain intermediate data 1 by using VR inference with VR = 15%. The second step was to obtain the intermediate data set 2 after sampling with a sampling interval of 60 s. The third step was to merge the intermediate data set 1 and the intermediate data set 2 in order to obtain the final output.

Table 1 shows that the original data set has 1800 DPs. After the inferencing of VR = 15% and 60 s interval sampling, there were 21 DPs left in the data set. In other words, 1800 − 21 = 1779 points were reduced. According to the formulas, SR = (1779/1800) × 100% = 98.83%; at the same time, AR = 95.95%.

**Figure 4:**
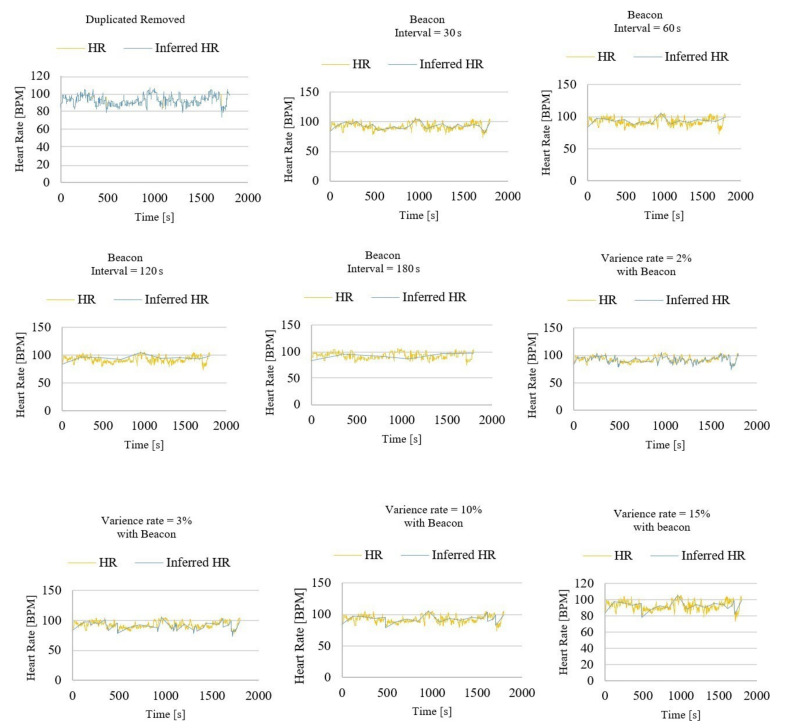
Evaluation of data inference framework (case 1 to case 9).

Figure 4 shows HR and inferred HR from case 1 to case 9. According to the experimental results, it is observed that, as the sampling interval increases, SR becomes larger, but AR becomes smaller. Moreover, as the value of VR becomes larger, SR becomes larger, but AR becomes smaller. In addition, when data inference is performed by combining time sampling (collecting beacon points) inference and VR inference, when the sampling interval is constant, the larger the VR, the more significant the AR improvement that is brought by the combined method.

Table 2 depicts the results of original data points of 6720 samples with heart rates being observed over 24 h captured every second. The results show that SR compromises AR, as SR increases when AR decreases due to higher AR requiring more DPs to process.

### 4.2. Privacy Preservation Evaluation

This section aims to study the efficiency of the proposed scheme from a privacy preservation perspective. Research on privacy-preserving approaches in eHealth clouds have commonly tended to focus on cryptographic methods, such as symmetric key encryption (SKE) and attribute-based encryption (ABE) [24,28]. In order to prove the efficiency of the proposed data inference framework for IoHT, the correlation between the plaintext size and crypto texts size can be tested in two main categories: SKE and ABE. For SKE evaluation, the simulation is conducted in OnlineDomain-Tools [29] for three main symmetric encryption techniques: advanced encryption standard (AES), data encryption standard (DES), and blowfish.

Over a course of 24 h in an experiment, a total of 1420 heart rate DPs were sensed and processed at various inference rates that ranged from 2.5%, 5%, 10%. and 20% VRs. After the inferencing algorithms are applied to the data, the number of DPs to be transferred were reduced significantly, as shown in Table 3. The VRs ranged from 0%, 2.5%, 5%, 10%, and 20%, which resulted in savings that ranged from 0%, 51.3%, 78.5%, 89.7%, and 98.8%, respectively. Plaintext size for 0% savings can be considered as 1024 B (1 MB). The plaintext size for other degree of savings can be obtained by:

(13) $$plaintext\;size\;\left(B\right)=\frac{\left(100-savings\right)\times 1024}{100}$$

**Figure 5:**
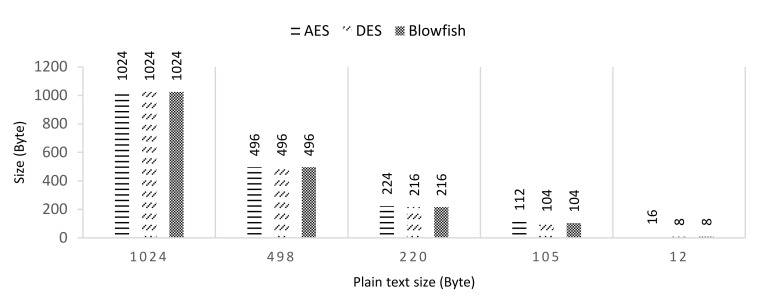
Evaluation of varying plain text size.

Figure 5 shows the impact of varying the plain text size. The mode is set to ECB (electronic code book) while maintaining the key at 128 and evaluating varying plain text sizes from 1024, 498, 220, 105, and 12 with AES, DES, and blowfish encryption functions. The results show that, as the size of the plain text size decreases, the size of crypto text also decreases accordingly. When comparing Table 3 and Figure 5, when VR is equal to 2.5%, the crypto text size for 1024 bytes of data is equal to 496 bytes. However, there is no clear evidence regarding whether one encryption technique was better than another. We have also evaluated the effectiveness of our solution by utilizing differential privacy. The dataset used in the experiment contains information regarding body temperature, gender, and heart rate for 130 people.

In the proposed model in this paper, data will eventually be used for statistical queries. For example, the average heart rate of someone in a day will be queried. The difference between one more record and one less record on the statistical results is defined as the sensitivity of the query algorithm, denoted as *∆f*. In order to provide E-differential privacy protection for our data, the output result will be:

(14) $$Out\_Result_{}=Real\_Result_{}+\;Laplace(\frac{\Delta f}{\varepsilon })$$

The Laplace (∆*f*/ε) is the Laplace noise which was added to protect the real data. According to Equation (14), the sensitivity of our query algorithm is first required to be analyzed, from which, an appropriate ε to obtain the required Laplace noise is selected. By definition of sensitivity, it is logical to infer that the greater the sensitivity, the greater the noise, and the smaller the sensitivity, the smaller the noise. An assumption is made that ∆*f* = 1 (that is, the addition of each new record will cause the result to change by 1, which is very large). Therefore, the following experiments are conducted with a sensitivity of 1 (∆*f* = 1), and the distribution of Laplace noise that is added to the data is equal to Laplace (1/ε). Noise is added in order to satisfy the Laplace (1/ε) distribution to each heart rate data in the original data set. Six experiments were performed, where ε was set equal to 0.01, 0.05, 0.1, 0.2, 0.5, 1.0, and the results are compared to observe how differential privacy protects the original data. Following this, the average values of the original data were identified and compared with the original data statistics in order to compare the performance of differential privacy.

**Figure 6:**
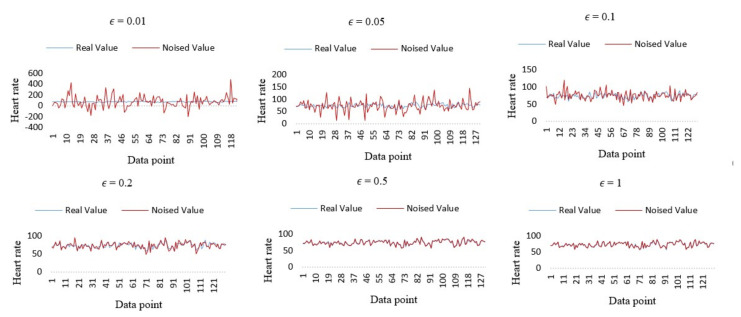
Evaluation of differential privacy with six different values of ε.

Figure 6 shows the experimental results under six cases of ε. The X-axis of each sub-figure shown in Figure 6 represents the index of the DP in the data set. The *Y*-axis represents the heart rate value of this DP. The blue line in the figure represents the heart rate value in the original data set, and the red line represents the value after the addition of Laplace noise to each DP in the original data. Lap (1/0.01), Lap (1/0.05), Lap (1/0.1), Lap (1/0.2), Lap (1/0.5), and Lap (1/1) are the distributions of Laplace noise added.

Based on the trend in changes of the sub-figures, it can be observed that, with the increase of *E*, the added noise begins to decrease i.e., the degree of deviation of red points from the blue line begins to decrease. When ε = 1, the noised data almost coincide with the original data. It can be observed that, when *E* is smaller (as in the sub-figure with ε = 0.01), the degree of privacy protection provided by random algorithms is greater, according to the trend of Figure 6. Conversely, when ε is larger (as in the sub-figure with ε = 1), the degree of privacy protection provided by random algorithms is lower.

Each DP, after adding Laplace noise, will deviate from the original data to a certain extent. However, this is not necessarily important, as users, in practice, may not query a specific value, such as their heart rate at a specific point, but may be more concerned about the average value over a certain period of time. The average value of both the original data set and the noised data set in all six experiments were calculated and the results are summarized in Table 3, which shows that the size of the noise added to the original data set is different in each experiment. The statistical results (after adding noise) deviate from the true statistical results (the raw data statistical results) to different degrees. The smaller the deviation, the higher the availability of data. When ε = 0.01, ε = 0.05, ε = 0.1, and ε = 0.2, there is a relatively large deviation level, and the availability of data is low. When ε = 0.5, the degree of deviation is very small (almost close to 0) and the data availability is high. The purpose of adding Laplace Noise is to ensure the availability of data while protecting user privacy. The experimental results shown in Figure 6 and Table 3 show that there is a compromise in privacy protection and data availability—obtaining greater results in one requires compromising the other. When comparing Figure 6 and Table 4 under these considerations, data protection capability and data availability were at their best when ε = 0.5.

## 5. Beneficial Applications

This section describes some possible applications for which the proposed inference solution could be implemented in mHealth and IoHT networks. There remains to be a myriad of possibilities for the use of healthcare big data in improving human lifestyle and wellbeing—these are just some of the examples.

### 5.1. Patient Monitoring of Disease Outbreak

It is crucial in disease outbreaks to quickly identify infected patients and potential carriers. mHealth technologies have the potential to identify individuals who may have been exposed to a disease, flagging those who may meet criteria to be considered for further testing or quarantine. Governments or agencies can use these data in order to more comprehensively inform population metrics, develop modelling, and to intelligently develop a public health response that can be objective and transparent to the public—avoiding the risk of generating panic. Educating the public will be key in public health responses to future disease outbreaks, and mHealth technologies with algorithms could allow for a localization of public health response to specific geographical areas of need.

### 5.2. Battery Conservation of Personal Health Devices

Some PHDs, such as pacemakers, operate on battery power, which requires a costly and invasive operation to replace. Therefore, conserving battery power is of importance to PHDs that are wireless and are implanted on or in the body. Previous research [20] found that battery power can be conserved with the use of an inference algorithm, while maintaining adequate data accuracy. As devices continue to develop with greater computational power, more complex algorithms can be applied for increasing their intelligence. A complex inference system that is applied on these devices could significantly reduce battery consumption without compromising data integrity.

### 5.3. Health Data for Identificationes

Biometrics, such as voice recognition or fingerprints, have been used in various applications for authentication. However, this cannot be used in remote applications. Health data could have use for user identification purposes, as privacy is a key requirement in eHealth and IoHT technologies. Whilst one aspect of health data, such as heart rate, may provide no identifying information, it could, in combination with others, represent a unique pattern that is specific to an individual, especially as a trend over time, and therefore risk breaching a user’s identity. The major expected outcomes for such an application could include (1) assessment of health data traits with measurable and standardized accuracy, (2) building a model of structured attributes that can affect the effectiveness of the health data being used for identification.

## 6. Conclusions

Energy efficiency and privacy preservation of sensitive health data are essential in IoHT networks, which largely consist of smart devices limited by battery constraints. In this paper, a two-tier data inference framework has been proposed in order to conserve energy consumption by reducing unnecessary data transmission within the IoHT network while still maintaining high accuracy. The results suggest that applying 1% to 2.5% variance rate by the inference system achieved the best accuracy. It was also shown that this amount of VR decreases nearly half of the crypto text size while using the main symmetric encryption techniques. Another major finding was that applying differential privacy with a *E* = 0.5 satisfies the data protection and data availability requirements. The experimental results show that the proposed system is beneficial for saving the energy of IoT devices and security analysis suggests that the differential privacy technique can protect against sensitive health data from being obtained maliciously. In our future work, we will investigate how to incorporate highly efficient blockchain and federated learning techniques [19,30] into our solution in order to improve privacy preservation while maintaining high accuracy in the data inferencing system.

## Figures and Tables

**Table 1:** Efficiency and accuracy evaluation for different cases.

Cases	Evaluation Condition	DP	Savings (%)	Accuracy (%)
Case 0	Original data	1800	N/A	N/A
Case 1	Removed duplication	1716	4.67	99.74
Case 2	Beacon Interval = 30 s	31	98.27	96.26
Case 3	Beacon Interval = 60 s	16	99.11	95.73
Case 4	Beacon Interval = 120 s	9	99.50	94.18
Case 5	Beacon Interval = 180 s	6	99.66	94.21
Case 6	Variance Rate (2%) with Beacon Interval = 60 s	182	89.88	97.57
Case 7	Variance Rate (3%) with Beacon Interval = 60 s	64	36.44	36.04
Case 8	Variance Rate (10%) with Beacon Interval = 60 s	22	98.78	96.14
Case 9	Variance Rate (15%) with Beacon Interval = 60 s	21	98.83	95.95

**Table 2:** Large Sample of Inference Rate for 24 h HR data (seconds) to compare variance rates (VR) related to Savings and Accuracy Rates.

Inferred rate	0	1.5%	2%	3%	5%	10%	15%
Data points	6720	4000	3559	3002	2074	1055	587
Savings (%)	0	40.7	47.0	55.3	69.1	84.3	91.2
Accuracy (%)	N/A	98.3	97.0	97.2	95.6	90.5	86.6

**Table 3:** Data savings for different values of variance rate (24 h samples).

VR	0%	2.5%	5%	10%	20%
DP	1420	691	306	146	17
Saving (%)	N/A	51.3	78.5	89.7	98.8

**Table 4:** Query results under different values of ε.

The value of ε	0.01	0.05	0.1	0.2	0.5	1.0
Average value of raw data	73.76	73.76	73.76	73.76	73.76	73.76
Average value of modified data	73.84	73.18	73.14	73.89	73.76	73.76

## Data Availability

The data presented in this study are available within the article.
